# Exploring Patient–Clinician Trust During Pregnancy Care: A Qualitative Study With Latine Patients

**DOI:** 10.1097/og9.0000000000000006

**Published:** 2024-04-30

**Authors:** Rose L. Molina, Maria Bazan, Giuliana Rivera Casul, Yessamin Pazos Herencia, Margarita Alegría, Leonor Fernandez, Maria Edelen, Anjali J. Kaimal, Michele R. Hacker, Jeanne-Marie Guise

**Affiliations:** Department of Obstetrics and Gynecology and the Department of Medicine, Beth Israel Deaconess Medical Center, Harvard Medical School, Departments of Medicine and Psychiatry, Massachusetts General Hospital, Department of Surgery, Brigham and Women's Hospital, Boston, Massachusetts; Universidad Científica del Sur, Lima, Peru; and Department of Obstetrics and Gynecology, University of South Florida, Tampa, Florida.

## Abstract

**OBJECTIVE:**

To explore patient experiences and perspectives regarding trust in clinicians during pregnancy care among Latine patients, focusing on those with Spanish as their preferred language.

**METHODS:**

We conducted a qualitative focus group study to explore trust in pregnancy care clinicians. Eligible participants self-identified as Hispanic/Latine, reported Spanish or English as their preferred language, were pregnant or had given birth in the previous year, and were at least 18 years old. Participants completed a brief demographic survey. We report descriptive statistics for survey responses. A semistructured discussion guide was developed from a literature review and feedback from a patient advisory panel. We report emerging themes using a modified grounded theory approach with an analysis of focus group transcripts. We mapped themes to the five dimensions of trust according to the Building Trust Initiative—communication, caring, comfort, cost, and competency—and barriers to trust.

**RESULTS:**

We conducted eight focus groups (five in Spanish, three in English) through Zoom between July and September 2023. Cost was not discussed in any of the focus groups. Rather, there was an emerging theme around *acompañamiento* (accompaniment is a distinct form of companionship in which there is shared power during a shared journey), particularly in the Spanish focus groups. Barriers to building trust exist at the interpersonal and health system levels, including miscommunication, lack of continuity, and brevity of appointments.

**CONCLUSION:**

This study explores how trust is conceptualized in pregnancy care according to the perspectives of Latine patients. We found that *acompañamiento* is a unique and crucial dimension of trust among Spanish-speaking patients.

## Abstract 2

In addition to communication, comfort, caring, and competency, accompaniment is a unique dimension of trust in pregnancy clinicians among patients whose preferred language is Spanish.

Trust among patients, clinicians, and health care systems is critical to achieve the quintuple aim of health care delivery: better health outcomes, better patient experiences, health equity, reduced costs, and clinician well-being.^1^ Research on building trust in health care is a key priority championed by national organizations, including the American Board of Internal Medicine Foundation and AcademyHealth.^2,3^ Trust is associated with higher quality of life and satisfaction with care.^4^ In contrast, mistrust is associated with underutilization of health services.^5^ Mistrust stems from a variety of negative experiences in health care, including discrimination,^6^ which may affect health-seeking behavior, especially among minoritized populations. Despite the centrality of trust in patient–clinician relationships, 50 years of trust research has revealed conceptual ambiguity in defining and measuring trust.^7^ In addition, very few studies examine the dynamics of trust—how it can be built, eroded, broken, and repaired over time.^7^ Pregnancy is an ideal window to understand better how patient trust in clinicians may change over time, especially among minoritized Latine patients who speak Spanish.

Language and cultural differences between patients and clinicians can threaten trust building. Language differences hamper every dimension of high-performing health systems: access, safety, quality, equity, patient experience, and cost.^8^ In the United States, 37 million people speak Spanish as their primary language.^9^ Studies show that patients with limited English proficiency experience less access to care,^10^ more adverse events,^11^ and worse quality of care^12^ than English-proficient patients. Recent studies have shown negative experiences of care, including mistreatment and discrimination among Hispanic/Latine individuals,^13^ especially those with Spanish as their primary language.^14^ Significant barriers to using qualified interpreters persist.^15–17^ Spanish-speaking patients express a strong preference for bilingual clinicians because of greater privacy, accuracy of communication, and sense of trust.^18,19^

This study aimed to explore patient experiences and perspectives regarding trust in clinicians during pregnancy care, specifically among patients with Spanish as their preferred language, a population known to experience limited access to health insurance^20^ and inequitable engagement in research compared with groups with English language preference.^21,22^

## METHODS

We conducted a qualitative focus group study to explore trust in clinicians during pregnancy care among patients with Spanish and English language preferences. We applied a grounded theory approach^23^ to explore how patients define trust in pregnancy care clinicians. We also identified facilitators and barriers to building trust and the dynamics of trust over time.

An interdisciplinary team of clinicians, qualitative researchers, behavioral scientists, and research trainees led this study. The focus group facilitator (M.B.) is a native Spanish-speaking physician trained in qualitative research with no prior relationship with participants. The two coders (M.B. and G.R.C.) identify as Latina physicians and are native Spanish speakers. They met regularly throughout the coding process and discussed the interplay of race, culture, ethnicity, and regional differences in the Spanish language that may have influenced the interpretation of findings. The coders debriefed regularly with the principal investigator to examine any potential biases in the analysis. The principal investigator (R.L.M.) is biracial with advanced professional proficiency in Spanish. The professional experiences of the principal investigator, a clinician caring for a large Spanish-speaking immigrant population, motivated this study.

We identified eligible participants through pregnancy appointment schedules, birth logs, and electronic medical records at Beth Israel Deaconess Medical Center. Inclusion criteria were as follows: self-identified as Hispanic/Latine, reported Spanish or English as their preferred language, were pregnant or had given birth in the previous year, and were at least 18 years old. Our primary interest was to explore the perspectives of Spanish speakers. We included English-speaking Latine patients to explore whether they expressed similar themes.

Eligible participants were mailed a letter inviting them to participate in focus group discussions in Spanish or English. Participants could either sign up for or decline to participate in the study. If participants did not respond within a week of the expected receipt of the recruitment letter, a research team member reached out to them by phone or during routine pregnancy or postpartum appointments to discuss the study and to review the consent. Participants were compensated with a $50 gift card for their participation.

Participants were asked to complete a brief demographic survey at the start of each focus group. The survey included 10 questions about age, ethnicity, country of origin, length of time living in the United States, language preference, and level of English proficiency. The Brief Acculturation Scale for Hispanics score was used to assess their self-reported English level and level of acculturation.^24^ Reasons that prevented participants from building trust with their clinicians were collected. Survey data were collected using REDCap (Research Electronic Data Capture).^25^

We developed a semistructured discussion guide based on a literature review about trust domains (Appendices 1 and 2, available online at http://links.lww.com/AOG/D648). Topics included defining trust in pregnancy care clinicians and care teams, how trust is built or eroded over time, external factors, and previous experiences that affect developing trust. These topics and discussion questions were refined in consultation with a patient advisor and a cultural broker who were recruited through previous related studies and the principal investigator's professional network. Eight focus groups were completed through Zoom from July to September 2023 (five focus groups were in Spanish, and three were in English), audio-recorded, and transcribed with professional transcription software. The Spanish transcripts were also translated into English with professional translation software and checked for accuracy by the bilingual research team members. The Spanish focus groups were completed first to ensure inclusion of voices often left out of research studies because of language barriers. M.B. was the facilitator, and Y.P. was the notetaker in all focus group discussions. The research team discussed emerging themes on a weekly basis, and saturation was reached after eight focus groups.

After completing the focus groups, two bilingual investigators (M.B. and G.R.C.) independently coded transcripts using Dedoose 7.5.9. An initial codebook was developed that was based on the key topics discussed. Both investigators performed open coding of transcripts in Spanish and English (according to the language in which the focus groups were held) through a constant comparative method whereby codes were iteratively refined into themes. The principal investigator (R.L.M.) resolved disagreements or areas of uncertainty. Emerging themes were mapped to the American Board of Internal Medicine Foundation's five dimensions of trust: communication, caring, comfort, competency, and cost.^3^ We applied the American Board of Internal Medicine Foundation framework for trust because that foundation and AcademyHealth have brought together a community of researchers to advance the field of trust research in health care.^2^

An advisory panel of two Spanish-speaking patients and a cultural broker reviewed the emerging themes of the focus group discussions and provided helpful feedback in triangulating the findings. Descriptive summary statistics were calculated for the survey responses. We report illustrative quotations in the tables in their original language to maintain the authenticity of participant voices. The study was approved by the Beth Israel Deaconess Medical Center Committee on Clinical Investigations (protocol 2023P000479).

## RESULTS

We identified 217 eligible participants, of whom 33 consented to participate in the eight focus groups (two–seven participants per group, lasting 35–53 minutes). We summarize the demographics in Table 1. Most participants were 25–34 years old (58.8%), with a mean age of 31.2 years (SD 5.2 years). Most participants had a self-reported English-speaking level of “not well” (38.2%), with Spanish only as the language most used to communicate at home (50.0%). When asked about reasons preventing participants from trusting their clinicians, 41.2% answered “not applicable,” and job insecurity and having a previous negative health care experience were the most common responses (17.6%). Table 2 has the English translations of the Spanish quotes.

**Table 1.** Participant Demographics (N=34)*

Characteristics	
Age (y)	31.2±5.2
18–24	4 (11.7)
25–34	20 (58.8)
Older than 35	9 (33.3)
Country of origin	
Dominican Republic	11 (33.3)
United States	5 (14.7)
Colombia	4 (11.8)
Guatemala	3 (8.8)
Venezuela	2 (5.9)
Honduras	2 (5.9)
El Salvador	1 (2.9)
Bolivia	1 (2.9)
Ecuador	1 (2.9)
Peru	1 (2.9)
Puerto Rico	1 (2.9)
Missing	1 (2.9)
Self-reported level of English spoken	
Excellent	8 (23.5)
Well	5 (14.7)
Not well	13 (38.2)
Not at all	7 (20.6)
Language used by participant to communicate at home	
Spanish only	17 (50.0)
Spanish more than English	7 (20.5)
Spanish and English equally	3 (8.8)
English more than Spanish	3 (8.8)
Only English	2 (5.9)
Language other than English or Spanish	1 (2.9)
Language used by participant to communicate with friends	
Spanish only	19 (55.9)
Spanish more than English	5 (14.7)
English more than Spanish	3 (8.8)
Spanish and English equally	2 (5.9)
Only English	3 (8.8)
Language other than English or Spanish	1 (2.9)
Language used by participant to read	
Spanish only	14 (4.1)
Spanish more than English	8 (23.5)
English more than Spanish	4 (11.8)
Spanish and English equally	5 (14.7)
Only English	1 (2.9)
Language other than English or Spanish	1 (2.9)
Language used by participant to think	
Spanish only	14 (41.2)
Spanish more than English	8 (23.5)
English more than Spanish	3 (8.8)
Spanish and English equally	3 (8.8)
Only English	3 (8.8)
Language other than English or Spanish	1 (2.9)
Missing	1 (2.9)
Reasons that prevented participants from trusting their clinicians^†^	
Job insecurity	6 (17.6)
Previous negative health care experience	6 (17.6)
Housing insecurity	3 (8.8)
Legal concerns	3 (8.8)
Food insecurity	0 (0.0)
Other	3 (8.8)
Not applicable	14 (41.2)

Data are mean±SD or n (%).

* There were 34 responses from 33 participants, because one participant joined two different focus groups.

† More than one response was possible.

**Table 2.** English Translations of Illustrative Quotes of Trust Dimensions and Facilitators

Spanish Quote	English Translation
Communication	
“*Yo creo que la comunicación entre ambos sea fluida, más que todo para poder despejar mis dudas y poderme sentir tranquila yo de lo que yo quiera, no de lo que yo necesito, de lo que yo quiera. De repente algunas preguntas que podamos tener, que son muchas porque soy mamá primeriza, entonces siempre tenemos muchas dudas, siento que la comunicación es lo principal.*”	“I believe that communication between us should be fluid, mainly to clarify my doubts and to make me feel at ease with whatever I want, not just what I need. There might be some questions that we could have and there are many because I am a first-time mom, so we always have a lot of doubts. I feel communication is the most important.”
Caring	
“*Yo pienso que la empatía, que antes de ser médicos sean personas, porque hay médicos que ya están acostumbrados a ciertas situaciones y son fríos.*”	“I think that empathy is important, that before being doctors, they should be human beings, because there are doctors who are already used to certain situations and can be cold.”
“*Yo pienso que la empatía es algo muy importante y la primera impresión también. Cuando uno va la primera vez, tú tienes muchas expectativas. Tienes muchas dudas y la forma como te reciba el profesional de salud va a determinar qué tanta confianza vas a tener tú para contarle todo, o vas a ir o como que limitado, como con pena, o con cierto temor.*”	“I believe that empathy is something very important, and the first impression as well. When you go for the first time, you have many expectations. You have many doubts, and the way the health care professional receives you will determine how much confidence you will have to tell them everything. You may either go with an open willingness to share or feel limited, hesitant, or even fearful.”
“*Que se preocupen por la salud de uno, que estén mandándome un mensaje o una llamada para ver si me siento mejor, que pregunten si estoy bien por el problema al que acudí antes, si estoy tomando la medicina.*”	“That they care about one's health, sending me a message or making a call to see if I feel better, following up on how I'm doing with the problem that prompted my previous visit, and checking if I'm taking the medicine.”
“*Que tu proveedor se preocupe, que tú sientas que en realidad se está preocupando por tu salud, que no sólo está cumpliendo con su trabajo o con su horario, sino que sientas que en realidad se está preocupando por cómo tú estás, que no eres alguien más”*	“That your provider cares, that you feel that they are genuinely concerned about your health, that they are not just fulfilling their job or schedule, but that you feel that they are truly concerned about how you are, that you are not just another person.”
Comfort	
“*Se nota la disposición del profesional, porque he ido a lugares que tratan de buscar la solución al problema rápido y otros como que te olvidan. Entonces, es bueno porque te hacen sentir que uno es importante, que el problema de uno es importante para ellos y están trabajando para buscar una solución al problema*”	“The provider's willingness is noticeable because I have been to places that try to find a quick solution to the problem, and others seem to forget about you. So, it's good because they make you feel that you are important, that your problem matters to them, and they are working to find a solution to it.”
“*Yo considero que sería la mejor opción poder confiar en tu proveedor porque cuando uno llega a confiar en esa persona, siento que uno puede expresarse mejor y poderle contar la situación de uno.*”	“I consider that it would be the best option to be able to trust your provider because when one comes to trust that person, I feel that one can express oneself better and be able to share one's situation with them.”
“*Ellos [los médicos] me atendieron muy bien y yo sentí como era como una familia y ellos me daban comida y atendían hasta a mis otros hijos que ya son adultos, incluso a mi esposo también le daban comida. Eran bien buenos*”	“They [the doctors] treated me very well, and I felt like it was a family. They provided me with food and took care of even my other adult children, and they also gave food to my husband. They were really nice.”
Accompaniment	
“*Entonces como el ir un poco más allá de lo que en realidad solo necesitas como mujer embarazada. Porque hay muchas cosas que te pueden ayudar en ese proceso y sí, te dan mucha información y te dicen ‘Mira esto es’ y te entregan un folleto y te dicen ‘Aquí está todo’ y ya. Pero en realidad se trata de ese acompañamiento que tu sientas.*”	“So, it's about going a little beyond what you actually just need as a pregnant woman. Because there are many things that can help you in that process, and yes, they give you a lot of information like 'look, this is it,' and they hand you a brochure, and okay, here's everything. But actually, it's about that support that you feel.”
“*No es crear una relación y que va a ser tu mejor amigo, pero sí, que tú puedas tener esa relación, porque al final es la persona que te va a estar acompañando durante nueve meses, entonces tú necesitas como crear esa cercanía. Es importante poder llamarlo inmediatamente, sea por WhatsApp, o por tal vez un correo electrónico o que te responda inmediato la llamada.*”	“It's not about creating a relationship as if they're going to be your best friend, but yes, you should be able to have that relationship because, in the end, that is the person who will be accompanying you for 9 months. So, you need to establish that closeness. It's important to be able to call them immediately, whether it is through WhatsApp, email, or getting an immediate response to a phone call.”
Competency	
“*Yo creo que también que sea una persona con la experiencia necesaria, capaz, que no sea alguien que esté aprendiendo o ensayando con una persona, que sea una persona que tenga la experiencia que uno anda buscando, y eso genera confianza.”*	“I also believe that it should be a person with the experience needed, capable, not someone who is learning or practicing with someone, but rather a person who has the experience that one is seeking, and that builds trust.”

**Fig. 1.**
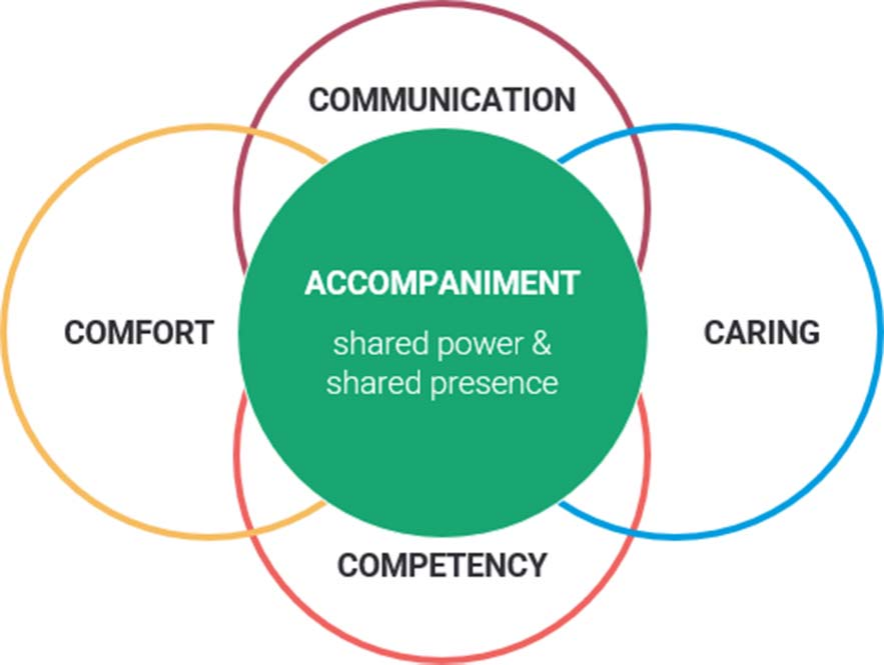
Trust dimensions during pregnancy care.

Participants in the Spanish and English focus groups described trust in their pregnancy care in two broad categories. First, clinician attributes and behaviors included providing clear communication, being welcoming and nonjudgmental, and being attentive and following up. Second, patient feelings and experiences were foundational for cultivating trust, which meant feeling cared for and comfortable expressing concerns. We present the findings according to the five dimensions of trust of the American Board of Internal Medicine Foundation. Notably, cost was not a salient theme. Rather, *accompaniment*—defined as companionship during a journey—was identified as an important dimension of trust, especially in the Spanish focus groups (Fig. 1). Overall, there was concordance in themes in both the Spanish and English groups, with only a few differences noted here.

*Molina. Patient–Clinician Trust During Pregnancy Care. O&G Open 2024*.

Trustworthy communication was clear, direct, honest, responsive to concerns, and timely. In response to a question about what builds trust, one participant described the following:

I believe that communication between us should be fluid, mainly to clarify my doubts and to make me feel at ease with whatever I want, not just what I need. There might be some questions that we could have and there are many because I am a first-time mom, so we always have a lot of doubts. I feel communication is the most important.

Additional participants emphasized the importance of feeling that their concerns are validated and that they are engaged in shared decision making in their pregnancy care. Another participant explained how the different modes of communication—written, verbal, and electronic—were all helpful and contributed to a sense of comfort during the visits:

For me it was the communication as well, making sure I had physical copies of appointments, even just emails they would send with tips for breastfeeding and all those kinds of tips. Just making me feel comfortable in general during the appointments was great. Just saying “feel free to do this,” “feel free to do that” rather than “you have to do it this way.”

Clinicians who demonstrated kindness, empathy, and warmth were described as critical for cultivating trust. One participant described the importance of empathy as follows: “I think that empathy is important, that before being doctors, they should be human beings, because there are doctors who are already used to certain situations and can be cold.” Multiple participants also explained the critical moment of when the clinician first enters the examination room and how that moment can either build or hinder trust:

I believe that empathy is something very important, and the first impression as well. When you go for the first time, you have many expectations. You have many doubts, and the way the health care professional receives you will determine how much confidence you will have to tell them everything. You may either go with an open willingness to share or feel limited, hesitant, or even fearful.

Participants described clinicians who demonstrated patient-centered actions as important for building trust. One of the most commonly mentioned facilitators was checking in and following up with patients to make them feel cared for: “…that they care about one's health, sending me a message or making a call to see if I feel better, following up on how I'm doing with the problem that prompted my previous visit, and checking if I'm taking the medicine.”

A participant expressed, “…that your provider cares, that you feel that they are genuinely concerned about your health, that they are not just fulfilling their job or schedule, but that you feel that they are truly concerned about how you are, that you are not just another person.” This sentiment indicates the importance of the patient feeling as though the clinician has concern for them as an individual, not just as a task to be completed.

Participants from both English and Spanish focus groups mentioned specific actions that clinicians can do that make patients feel comfortable, which is foundational for cultivating trust. For example, clinicians who remember details about the previous appointment create a welcoming environment that builds comfort and trust. One participant said:

It kind of comes down to how comfortable I feel around the person…I'll feel more comfortable if they seem like more human, if they sort of like level with me…if they show me empathy and if they show me kind of that respect and care by remembering things about me. My nurse practitioner, for example, always remembered the name of my toddler during my second pregnancy. She always remembered to ask about how he's doing. There were little things when she was just really human and friendly that helped us to build a relationship.

Another participant cited the brevity of some encounters, which made her feel forgotten and that her concerns were not important:

The provider's willingness is noticeable because I have been to places that try to find a quick solution to the problem, and others seem to forget about you. So, it's good because they make you feel that you are important, that your problem matters to them, and they are working to find a solution to it.

Participants also described how having trust in a clinician allows them to share more openly about their concerns: “I consider that it would be the best option to be able to trust your provider because when one comes to trust that person, I feel that one can express oneself better and be able to share one's situation with them.”

One difference between the Spanish and English focus groups was how each explained the feeling of comfort. Participants in the Spanish groups mentioned the concept of *sentirse en familia* (feeling like family) as a critical foundation for building trust. One participant described this as follows: “They [the doctors] treated me very well, and I felt like it was a family. They provided me with food and took care of even my other adult children, and they also gave food to my husband. They were really nice.” This degree of familiarity and familialism was not explored to the same degree in English groups. However, participants in English groups did emphasize the importance of feeling comfortable and at ease with their clinicians. They described that feeling comfortable involves having the space to share other factors that are on their minds and may not be directly related to pregnancy. One participant mentioned, “I guess it goes to that holistic aspect because my provider created that space, and I was intentional about bringing in other aspects of my life that affected my pregnancy. We were able to have an outcome that was right for me.”

In addition, participants from both language groups stated that “going the extra mile” (*ir más allá*) plays a role in establishing trust with clinicians. We identified a salient theme around accompaniment in the Spanish focus groups, which was not named in the English focus groups. Accompaniment is a distinct form of companionship in which there is shared power during a shared journey. One participant described:

So, it's about going a little beyond what you actually just need as a pregnant woman. Because there are many things that can help you in that process, and yes, they give you a lot of information like “look, this is it,” and they hand you a brochure, and okay, here's everything. But actually, it's about that support that you feel.

Another participant described the closeness with one's clinician that is foundational to the concept of accompaniment:

It's not about creating a relationship as if they're going to be your best friend, but yes, you should be able to have that relationship because, in the end, that is the person who will be accompanying you for 9 months. So, you need to establish that closeness. It's important to be able to call them immediately, whether it is through WhatsApp, email, or getting an immediate response to a phone call.

Other participants described this proximity as a friendship in which their clinician is always there for them, no matter what arises during the pregnancy journey.

Lastly, some participants recognized the importance of clinician expertise and demonstrated competency in pregnancy care. One participant shared: “I also believe that it should be a person with the experience needed, capable, not someone who is learning or practicing with someone, but rather a person who has the experience that one is seeking, and that builds trust.” Although this dimension of trust was discussed in both Spanish and English groups, competency was explored less often than the other dimensions above.

**Fig. 2.**
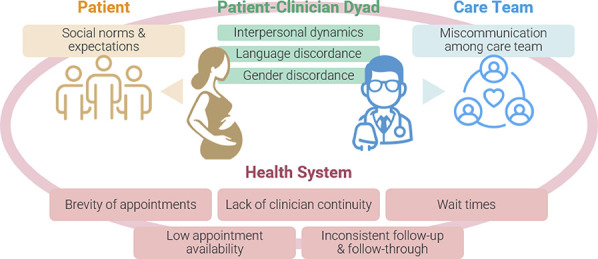
Barriers to trust.

Participants described barriers to building trust on two levels: at the interpersonal level with their clinicians and at the health system level (Fig. 2). At the interpersonal level, participants shared extensively about miscommunication (Table 3). One participant described: “A doctor was telling me one thing, and another was telling me something else, so I didn't know what to do. But what could I do? All I could do was just to stay with them, but that didn't seem right to me.” Hearing contradictory information from the medical team poses barriers to developing a sense of trust in the individual clinician or the care team. Another dimension of miscommunication is navigating language barriers even with qualified interpreters. One participant described how she used her limited English skills to identify when an interpreter did not correctly interpret what the doctor had said: “Sometimes, interpreters think that one doesn't understand what they are saying, but one does. So, the doctor might have meant one thing, and the interpreters say something completely different, and it was serious. At that moment, I felt it was a very bad experience.” Inaccurate interpretation and not feeling comfortable enough to share their concerns in detail were salient themes: “It's uncomfortable because sometimes you want to express certain concerns, certain fears, and you have to speak limitedly through the interpreter. So, you limit yourself in expressing all of that. If it had been with people directly speaking the same language, well, you explain everything, and that way we build more trust.” A few participants cited gender discordance with their clinicians as an important barrier in building trust.

*Molina. Patient–Clinician Trust During Pregnancy Care. O&G Open 2024*.

**Table 3.** English Translations of Illustrative Quotes of Barriers to Building and Rebuilding Trust

Spanish Quote	English Translation
Interpersonal barriers	
“*Una doctora me decía una cosa y la otra me decía otra cosa, entonces yo no sabia que hacer, pero que podía hacer? Lo único que hacía nada más era seguir con ellas, pero eso no me pareció.*”	“A doctor was telling me one thing, and another was telling me something else, so I didn't know what to do. But what could I do? All I could do was just to stay with them, but that didn't seem right to me.”
“*A lo mejor en el proceso de entre una transición de un doctor y otro, que te expliquen: 'Bueno, ya no va estar este doctor, pero va a estar esta doctora o doctor'. Entonces como que la transición se realice de manera amigable. No que llegues el día de la consulta y sea otro doctor y no te lo expliquen. Yo creo que en caso sea necesario que haya un cambio, la transición es muy importante para poder entablar otra vez esa confianza.*”	“Maybe in the process of transitioning from one doctor to another, they explain to you: 'Well, this doctor won't be here anymore, but this doctor will be here' So that the transition takes place in a friendly manner. It's not like you arrive on the day of the appointment and there's another doctor without any explanation. I believe that in case a change is necessary, the transition is very important to be able to establish that trust again.”
“*A veces los intérpretes piensan que uno no entiende lo que ellos dicen y uno si entiende. Entonces de pronto el doctor quería decir una cosa y los intérpretes dicen algo totalmente distinto y fue grave. Entonces en ese memento sentí que fue una muy mala experiencia.*”	“Sometimes, interpreters think that one doesn't understand what they are saying, but one does. So, the doctor might have meant one thing, and the interpreters say something completely different, and it was serious. At that moment, I felt it was a very bad experience.”
Health system barriers	
“*Uno llega y de pronto lo atiende muy rápido y no tienen el tiempo de sentarse a escucharlo. A veces también es complicado por el tema del idioma.*”	“One arrives, and suddenly they attend to you very quickly, without taking the time to sit down and listen to you. Sometimes it's also complicated due to language barriers.”
“*La atención es más personalizada [en Colombia]. Por ejemplo, en Colombia yo tenía acceso al celular de mi doctora y en cualquier memento podía escribirle aquí, en Estados Unidos, tienes que esperar a la cita.*”	“The care is more personalized in Colombia. For example, in Colombia, I had access to my doctor's phone, and I could message her anytime. Here in the United States, you have to wait for the appointment.”
“*Es incómodo porque a veces tú quieres expresar ciertas inquietudes, ciertos temores y tienes que hablar limitado con la intérprete, entonces tú te limitas a expresar todo eso. Si hubiese sido con unas personas directamente hablando el mismo idioma, pues uno le explica todo y así entramos más en confianza.*”	“It's uncomfortable because sometimes you want to express certain concerns, certain fears, and you have to speak limitedly through the interpreter. So, you limit yourself in expressing all of that. If it had been with people directly speaking the same language, well, you explain everything, and that way we build more trust.”
“*Yo tuve que ir a emergencia por un sangrado y le escribí a la doctora. La doctora me dijo que tenía que esperar porque debía aguardar hasta las 11 semanas, lo cual es bastante preocupante porque ya había tenido una mala experiencia de aborto. Entonces, aquí uno se siente entre la espada y la pared en ese sentido, ya que no se puede hacer nada. Pasé tres meses esperando la cita del ginecólogo.*”	“I had to go to the emergency room due to bleeding, and I wrote to the doctor. The doctor told me I had to wait because I had to wait until 11 weeks, which is quite concerning because I had already had a bad experience with a miscarriage. So, you feel caught between a rock and a hard place in that sense, as there's nothing that can be done. I spent 3 months waiting for the gynecologist appointment.”
“*Aquí la respuesta es cortante; allá en República Dominicana, te sientes más en confianza, más completa. Por ejemplo, si tienes dolor de cabeza, la doctora allá te explica y proporciona algo, mientras que aquí solo te recomiendan Tylenol.*”	“Here, the response is curt; back in the Dominican Republic you feel more at ease, more taken care of. For example, if you have a headache, the doctor there explains and provides something, while here, they just recommend Tylenol.”
Rebuilding trust	
“*Considero que tal vez sería algo difícil porque una vez que hayan hecho algo que me ha incomodado o molestado, yo trato de ver la manera de ya no de ir a ese lugar. Ya me queda esa desconfianza de que lo van a volver a hacer.*”	“I think that it might be somewhat difficult because once they have done something that has made me uncomfortable or upset, I try to find a way to no longer go to that place. I am left with that distrust that they will do it again.”

At the health system level, participants recounted the constraints that affected their ability to build trust with their clinicians (Table 3). For example, participants cited lack of appointment availability, wait times for appointments, brevity of appointments, and lack of consistent follow-up and follow-through with recommendations. Participants in both the English and Spanish focus groups discussed the lack of continuity with a single clinician as a significant barrier. However, they expressed the desire for clinicians to communicate this in advance, helping patients know what to expect in the following appointment:

Maybe in the process of transitioning from one doctor to another, they explain to you: “Well, this doctor won't be here anymore, but this doctor will be here.” So that the transition takes place in a friendly manner. It's not like you arrive on the day of the appointment and there's another doctor without any explanation. I believe that in case a change is necessary, the transition is very important to be able to establish that trust again.

One participant noted that the brevity of appointments is a particular barrier for individuals who speak languages other than English because of the added time needed for getting an interpreter and the extent of the conversation when an interpreter is present: “One arrives, and suddenly they attend to you very quickly, without taking the time to sit down and listen to you. Sometimes it's also complicated due to language barriers.”

Many participants discussed how their previous pregnancy experiences, whether in the United States or their country of origin, shaped their expectations of care, which were essential for building or eroding trust (Fig. 2). One consistent theme was the difference in direct access to doctors. One participant reported “The care is more personalized in Colombia. For example, in Colombia, I had access to my doctor's phone, and I could message her anytime. Here in the United States, you have to wait for the appointment.” Participants also noted the frequency of contact with the pregnancy care team during the first trimester. Many described that they struggled to get timely appointments in the first trimester in the United States, whereas they were attended to much more quickly in their countries of origin during those first 12 weeks. One participant shared:

I had to go to the emergency room due to bleeding, and I wrote to the doctor. The doctor told me I had to wait because I had to wait until 11 weeks, which is quite concerning because I had already had a bad experience with a miscarriage. So, you feel caught between a rock and a hard place in that sense, as there's nothing that can be done. I spent 3 months waiting for the gynecologist appointment.

Participants also noted that in the United States appointments are concise, not allowing enough time to address their concerns. In addition, they explained the difference in medication protocols between the United States and their home country during pregnancy: "Here, the response is curt; back in the Dominican Republic you feel more at ease, more taken care of. For example, if you have a headache, the doctor there explains and provides something, while here, they just recommend Tylenol." Such expectations regarding frequency of contact, direct method of communication, and access to appointments in a timely way shape patient experiences and trust.

Rebuilding trust when broken was feasible for some participants in the English focus groups. Some ways to recover trust include acknowledging errors and putting in extra effort to rebuild trust:

For me, showing the interest of trying to rebuild that trust, so showing that they care and really making an effort. There's times where I have seen different physicians where it just feels very transactional. If I see the providers making an effort, even if it's just to take a little more time explaining something to you or providing you resources, giving you a certain website, or just making that little bit of an extra effort to make sure that you feel understood.

In contrast, some participants from the Spanish focus groups mentioned that trust is almost impossible to recover once it is broken: “I think that it might be somewhat difficult because once they have done something that has made me uncomfortable or upset, I try to find a way to no longer go to that place. I am left with that distrust that they will do it again.”

## DISCUSSION

This study explores the construct of trust between patients and clinicians during pregnancy among Spanish- and English-speaking Latine individuals. We mapped the themes to the five dimensions of trust according to the Building Trust Initiative. We found that some of those dimensions resonated strongly (communication, caring, and comfort) in both the Spanish and English groups. Competency resonated somewhat in both groups, and cost did not resonate at all with either group. Cost was likely less important because all pregnant individuals in Massachusetts who may not be eligible for other insurance qualify for the state's Medicaid program, which provides comprehensive coverage during prenatal, childbirth, and postpartum care.

Overall, the themes from the focus groups were concordant between the groups, with a few notable exceptions. The Spanish groups discussed the importance of feeling like family with their pregnancy care clinician, which was not addressed in the same way in the English groups. In addition, the Spanish groups explored *acompañamiento* (accompaniment) as a unique dimension of trust. Accompaniment has roots in liberation theology in Latin America. It has been championed by Dr. Paul Farmer, a medical anthropologist and advocate for social justice: “To accompany someone is…to go somewhere with him or her, to break bread together, to be present on a journey with a beginning and an end.”^26^ Farmer argued, “Yet accompaniment is not simply walking together. It requires recognizing real-world complexities, acknowledging the asymmetries of power and privilege, and being willing to address these while walking together.”^26^ Clinicians can accompany patients by sharing power and presence throughout pregnancy.

This study presents actionable recommendations to enhance trust between patients and clinicians at both the interpersonal and systems levels. At the interpersonal level, our findings highlight the importance of honest, transparent, and respectful communication and small acts demonstrating caring (eg, showing empathy and having a warm disposition and demeanor). These clinician behaviors create a sense of comfort among patients, who then develop trust in response to these behaviors. Although communication skills are often a significant competency in undergraduate medical education, these skills may erode over time and require continual reflection and practice, especially in the context of busy clinical schedules. One example of a team communication tool for labor and delivery is TeamBirth,^27^ which is being implemented across multiple states. However, TeamBirth was initially designed for English-speaking patients; additional work is needed to develop communication tools that are designed to bridge language barriers. Training in best practices for working with interpreters to connect with and build trust with patients across language and cultural barriers is also needed.

Although additional training and support for clinicians in demonstrating trustworthiness are needed, clinician behaviors are often influenced heavily by health systems constraints. For example, the brevity of prenatal care appointments limits the time available to engage authentically with patients with language-access needs. Health systems usually do not schedule additional time for interpreted visits. This time constraint places undue pressure on clinicians to move through the encounter as quickly as possible given the added time required for interpretation. Enhancing language access at all points of care—from scheduling to triaging concerns to the visit itself to following up on results and management plan—is necessary for patients with language barriers to develop trust in the clinician and in the broader health system.

Our study is unique in intentionally engaging Latine individuals with Spanish and English preferred languages. Few studies apply language as a critical lens for analysis and interpretation. Several themes differed by language group, underscoring how to build trust with each group. Language differences in describing patient experiences have essential implications for patient-centered research, including developing appropriate patient-reported outcomes in each language group rather than relying solely on translation from measures derived in English-speaking populations. Funding agencies should support researchers in developing studies that engage participants with different linguistic backgrounds.

The findings of this study must be considered in the context of its limitations. First, we did not seek to generate generalizable knowledge applicable to all populations. We acknowledge the vast diversity within the Latine population; our study did not yield complete representation of the communities of focus. Although we believe that the findings may be transferable to some populations during pregnancy care, we urge readers to consider the context of their priority populations and how these findings may be relevant in those settings. We also recognize that the authors’ positionalities influenced how we generated themes and interpreted the findings.

This study explores how trust is conceptualized, what facilitators and barriers enable or prevent building trust, and how trust may erode or be regained over time in the context of pregnancy care according to the perspectives of Latine patients. By centering the voices of a minoritized community, this study boldly challenges the paradigm of extrapolating findings from the majority to different minoritized groups, especially those with language barriers.
